# Intraperitoneal hemorrhage secondary to ruptured omental artery aneurysm associated with polyarteritis nodosa: a case report

**DOI:** 10.1186/s40792-023-01675-y

**Published:** 2023-06-02

**Authors:** Mohamed H. El-Farra, Rayan Yahia, Aysenur Cetinkaya, Nahidh Hasaniya

**Affiliations:** 1 University of California Riverside School of Medicine, 92521 Botanic Gardens Dr, Riverside, CA 92507 USA; 2 Istanbul Medipol University, Istanbul, Turkey; 3 Department of Surgery, St. Bernardine Medical Center, San Bernardino, CA USA

**Keywords:** Polyarteritis nodosa, Aneurysm, Omental artery

## Abstract

**Background:**

Polyarteritis nodsa (PAN) is a rare disease characterized by acute focal inflammatory damage to small and medium arteries. PAN complicated by ruptured aneurysm is an infrequent presentation with the most affected arteries being the renal and mesenteric arteries.

**Case presentation:**

A 76-year-old female presented with a low-grade fever, generalized body aches, and abdominal pain. Investigation revealed intraperitoneal bleeding secondary to a ruptured and actively bleeding right omental artery aneurysm. Clinical manifestation, angiography and histology were consistent with PAN. Laparotomy was performed for stabilization and resection of the bleeding aneurysm followed by post operative steroids and cyclophosphamide. Patient was discharged in a stable condition. We reviewed seven cases found in the literature of omental artery aneurysm and rupture. Four cases were proceeded with laparotomy and aneurysm resection while three cases were proceeded with a less invasive approach of arterial embolization.

**Conclusions:**

Omental artery aneurysm is a rare occurrence with even fewer reported cases associated with PAN. Of the seven reported cases, all patients were treated with a surgical intervention. In addition, PAN patients should be treated post-operatively with a course of steroids and cyclophosphamide.

## Introduction

Polyarteritis nodosa (PAN) is a rare form of vasculitis affecting approximately 30.7 per 1,000,000 people with a predominance for middle-aged men [1]. PAN is characterized by segmental transmural inflammatory damage to the musculature of small and medium-sized arteries [2]. The inflammation of these arteries often progresses to thrombosis, ischemia, and necrosis which ultimately weakens the vessel wall predisposing them to the risk of aneurysm formation and eventual rupture [3]. Due to the systemic deposition of immune complexes, PAN can present in a variety of forms. Constitutional symptoms can present as fatigue, weight loss, fever, and arthralgias. Aneurysm rupture is rare, but typically occurs in the renal, hepatic, or mesenteric arteries [3]. We report a rare case of PAN-associated omental artery aneurysm rupture.

## Case report

A 76-year-old female was admitted with 2 weeks history of low-grade fever and generalized body aches. Further inquiry revealed 3-day history of gradual diffuse abdominal pain. The pain was markedly worse in the right lower quadrant. The patient had no other past surgical or medical problems. She denied any alcohol, tobacco or drug abuse. In the emergency department the vital signs revealed temperature of 38.5 °C, blood pressure of 149/87 mmHg, pulse of 117 bpm, and respiratory rate of 22 breaths/min. Systemic physical exam revealed pale conjunctivae and systolic ejection murmur of 2/6 over the apex of the heart. Examination of the abdomen revealed mild distention with tenderness all over the abdomen that was worse in the right and lower abdomen. The rest of the examination was within normal limits.

Laboratory results revealed white blood cell count of 18,500 per mm^3^, hemoglobin of 9.5 gm/dL, platelet count of 175 per mm^3^, calcium level of 7.3 mg/dL, albumin level of 2.6 gm/dL, creatinine level of 0.93 mg/dl, and C-reactive protein level of 13.3 mg/dl. All other laboratory values were within normal limits.

**Fig. 1:**
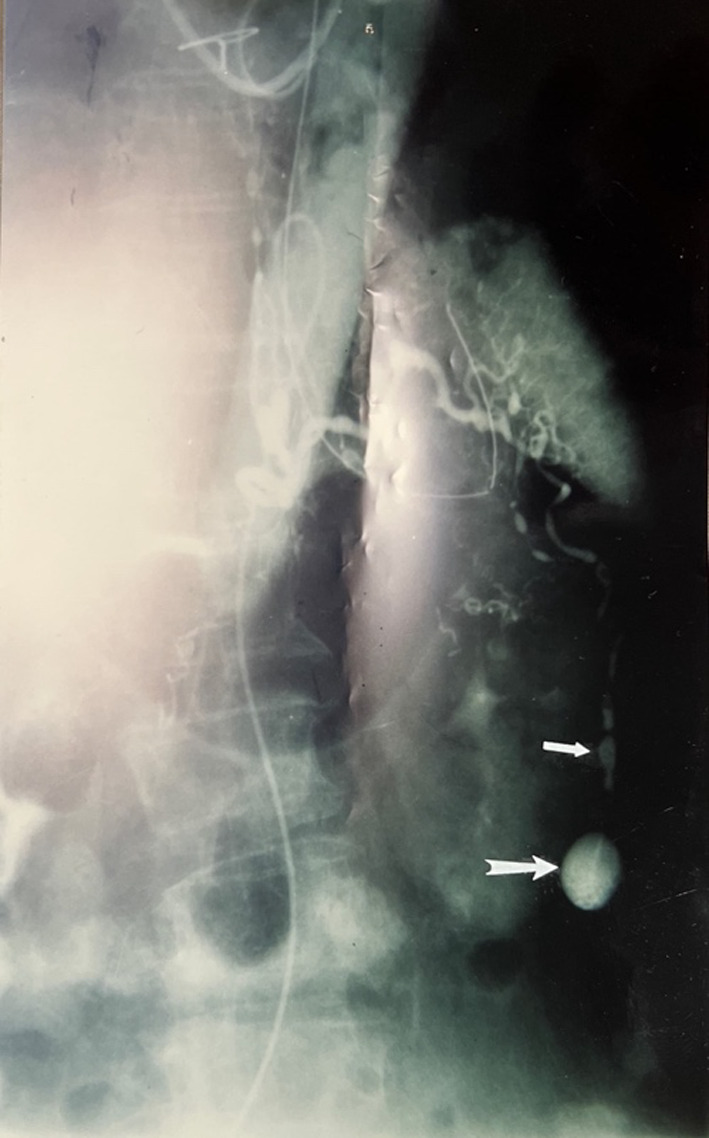
Selective celiac artery angiography showing several aneurysms (arrows)

**Fig. 2:**
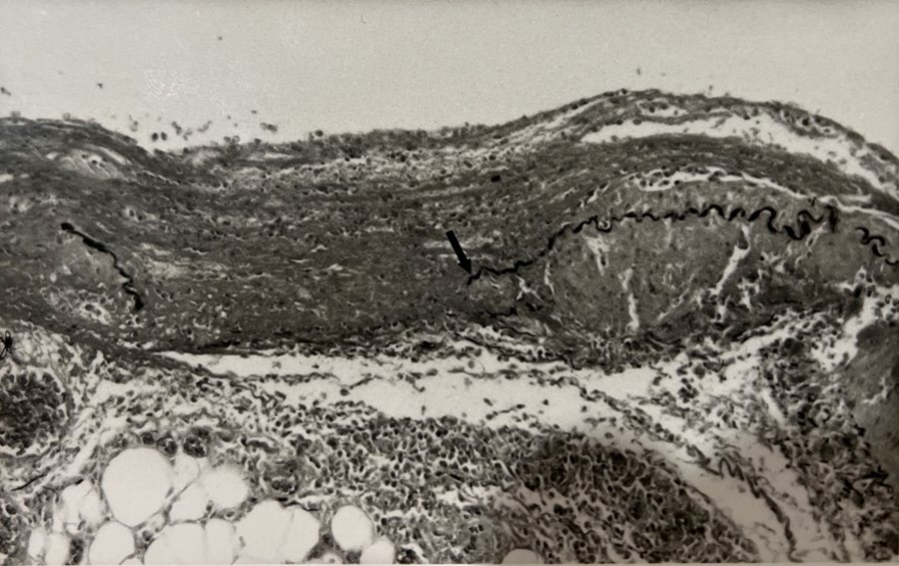
Histopathology shows necrotizing arteritis (Fibrinoid necrosis, polymorphonuclear leukocyte infiltrate, no fungus and disruption of the elastic membrane [arrow])

CT scan abdomen revealed intraperitoneal fluids. CT angiogram was unavailable, therefore, we proceeded with an abdominal aortic angiogram, which revealed a right omental artery aneurysm (Fig. 1). Laparotomy revealed a large bleeding omental artery in the right side of the abdomen. This was resected. Other unruptured aneurysms were seen but were not resected. Histopathology revealed full layer thickness arteritis with widespread neutrophils infiltration and necrotizing vasculitis confirming a diagnosis of PAN (Fig. 2). There was no evidence of fungal or bacterial elements in the vessel wall. Subsequent angiography revealed multiple aneurysms in the omental arterial system of the abdomen. The patient tolerated the procedure without complication and the abdomen was closed without problems.

Based on her histopathologic diagnosis of PAN the patient was started on intravenous steroids and cyclophosphamide postoperatively for systemic inflammation. During her hospitalization she had persistent low-grade fever; all her cultures were negative. After 10 days, the patient was discharged home in stable condition. Follow-up revealed a stable condition and no recurrence of her symptoms. Patient was lost for follow-up after 1 month.

## Discussion

PAN is a rare cause of vasculitis first described by Rokitansky in 1852 and published by Kussmaul and Maier in 1866 [4]. Primary etiology of PAN can be idiopathic; however, secondary causes have been linked to chronic Hepatitis B and C infection, Hairy Cell Leukemia, and Adenosine Deaminase 2 deficiency [5]. Due to the systemic deposition of immune complexes, PAN can present in a variety of forms. Multisystem involvement is common in this disease with the kidneys and GI system typically being affected. Other systems involved can include the liver, muscles, nervous, mesenteric vessels, and skin [6]. Therefore, other signs of PAN can include skin lesions, hypertension, neurologic dysfunction, myalgias, renal insufficiency, and abdominal pain. The lung is typically spared in PAN preventing the development of pulmonary symptoms.

Due to the rarity of the disease, clinical suspicion should be confirmed with a biopsy of the medium-sized arteries for histological analysis or angiography if biopsy cannot be performed [7]. Histological presentation of PAN is characterized by transmural inflammation of the arterial wall with fibrinoid necrosis and leukocytic infiltrates [8]. The inflammation of the arteries leads to thickening of the vessel wall and intimal proliferation causing luminal narrowing. Through this mechanism, the vessels are predisposed to thrombosis and the supplied tissue receives reduced blood flow leading to ischemia and necrosis. Over time, the wall of the vessels become weakened and promote aneurysm formation that may eventually lead to the rare complication of vessel rupture.

Visceral artery aneurysm is an extremely rare condition with a reported incidence of approximately 0.01–2% [9]. Previous cases in the literature have reported predominate locations were in the splenic artery (60%), hepatic artery (20%), celiac trunk (5.5%), superior mesenteric artery (4%), gastric and gastroepiploic arteries (4%), intestinal arteries (3%), and the pancreaticoduodenal arteries (2%). The etiology of visceral artery aneurysm is typically atherosclerosis occurring approximately 32% of the time, followed by medial degeneration (24%), abdominal trauma (22%), infection/inflammation (10%), and other causes such as connective tissue disorders, hyperflow conditions and vasculitis (12%) [10]. There have been very few documented cases of omental artery vessel aneurysm and rupture (Table 1). In addition, compiled with the rarity of its etiology due to PAN as seen in our case, there are even fewer reports in the literature of such a case.

**Table 1:** Compilation of found case reports of omental artery aneurysm rupture [11-17]

Case Report	Patient age/sex	Comorbidities	Chief symptoms	Management	Perioperative complications	Long-term complications
Jacobs et al.	63, M	Obesity, DM T2, Atrial fibrillation	Abdominal discomfort, Hypovolemic shock	Laparotomy, arterial ligation	None reported	None reported
Bettini et al.	73, F	CABG, Aortic valve replacement, Hypertension	Acute abdominal pain, Vomiting	Laparotomy, partial omentectomy	None reported	None reported
Park et al.	71, M	Continuous ambulatory peritoneal dialysis	Bloody peritoneal effluents, AMS, Hypovolemic shock	Laparotomy, omentectomy	None reported	None reported
Fernandes et al.	66, M	Atrial fibrillation	Epigastric pain, Hypovolemic shock	Laparotomy, arterial resection	None reported	None reported
Takahashi et al.	27, M	None reported	Acute upper abdominal pain, Anemia	Transcatheter arterial embolization (TAE)	None reported	None reported
Bundy et al.	74, M	None reported	LUQ and epigastric pain	Direct percutaneous embolization	None reported	None reported
Matsumoto et al.	25, M	None reported	LUQ pain	TAE, Partial omentectomy	None reported	None reported
El-Farra et al.	75, F	None	Diffuse abdominal pain	Laparotomy, arterial resection	None reported	None reported

Omental artery aneurysms are rare and are associated with a high risk of rupture necessitating prompt treatment. Due to its rarity definitive guidelines regarding indications for intervention or surgery have not yet been established. However, an open surgical approach has been most frequently reported. Kramman et al. reported an endovascular embolization approach that was successful 85% (11/13) of the time [18]. However, in more recent years, there has been a discussion of non-operative management with corticosteroids and cyclophosphamide, which decreases the aneurysm size and increases survival rates up to 80% [19, 20]. In our case, a combination of both open surgical resection and post-operative management with cyclophosphamide and corticosteroids were needed due to the nature of the patient’s urgent clinical presentation.

## Conclusion

The treatment of PAN vasculitis with a rare presentation of visceral artery aneurysm is achieved by either operative or non-operative management. Conservative treatment includes the use of corticosteroids and cyclophosphamide and is reserved for patients with non-urgent clinical presentations. Surgical interventions include aneurysm resection or embolization and is reserved for acute abdomens.

## Data Availability

Not applicable.

## Abbreviations

PAN: Polyarteritis nodosa
